# Pre-adolescent children exhibit lower aerosol particle volume emissions than adults for breathing, speaking, singing and shouting

**DOI:** 10.1098/rsif.2021.0833

**Published:** 2022-02-23

**Authors:** Mario Fleischer, Lukas Schumann, Anne Hartmann, Reuben Scott Walker, Liliana Ifrim, Dorothea von Zadow, Jonas Lüske, Joachim Seybold, Martin Kriegel, Dirk Mürbe

**Affiliations:** ^1^ Department of Audiology and Phoniatrics, Charité-Universitätsmedizin Berlin, Corporate Member of Freie Universität Berlin and Humboldt-Universität zu Berlin, 10117 Berlin, Germany; ^2^ Vorstand Krankenversorgung, Charité-Universitätsmedizin Berlin, Corporate Member of Freie Universität Berlin and Humboldt-Universität zu Berlin, 10117 Berlin, Germany; ^3^ Technische Universität Berlin, Hermann-Rietschel-Institut, 10587 Berlin, Germany

**Keywords:** aerosol particle emission, pre-adolescent children, voice

## Abstract

Speaking and singing are activities linked to increased aerosol particle emissions from the respiratory system, dependent on the utilized vocal intensity. As a result, these activities have experienced considerable restrictions in enclosed spaces since the onset of the COVID-19 pandemic due to the risk of infection from the SARS-CoV-2 virus, transmitted by virus-carrying aerosols. These constraints have affected public education and extracurricular activities for children as well, from in-person music instruction to children’s choirs. However, existing risk assessments for children have been based on emission measurements of adults. To address this, we measured the particle emission rates of 15 pre-adolescent children, all eight to ten years old, with a laser particle counter for the test conditions: breathing at rest, speaking, singing and shouting. Compared with values taken from 15 adults, emission rates for breathing, speaking and singing were significantly lower for children. Particle emission rates were reduced by a factor of 4.3 across all conditions, whereas emitted particle volume rates were reduced by a factor of 4.8. These data can supplement SARS-CoV-2 risk management scenarios for various school and extracurricular settings.

## 1. Introduction

The respiratory intake of virus-carrying particles is the primary source of transmission for SARS-CoV-2 viruses. Aerosol particles formed in the airways of infectious individuals can carry the SARS-CoV-2 virus, and through their release into the surrounding air, may be inhaled by others. In addition to their formation in the alveoli during breathing, aerosol particles are also generated in the airways by the articulators and the vibrating vocal folds during phonatory processes such as speaking, singing and shouting [1,2]. Considerably more aerosol particles are emitted during speaking when compared with breathing at rest, whereby the rate of emission depends substantially on the loudness of vocalization. Even higher particle emission rates have been recorded for singing when compared with speaking [3–6], both of which are surpassed by emission rates observed during shouting [7]. With higher emission rates, the risk of transmission increases within the near and far field of an infectious person in enclosed spaces through the increased concentrations of virus-carrying aerosol particles. Apart from emission rates, the risk of transmission depends on several further factors, including a prolonged stay of the infected person(s), insufficient ventilation and small dimensions of the space. For scenarios exhibiting these risk factors, e.g. collective singing over an extended choir rehearsal, higher rates of infection have been recorded [8–10]. The aerosol particle emission rates and the derived particle volume are therefore crucial factors for the risk assessment of exposure to SARS-CoV-2 and essential considerations for risk management.

This study aims to determine the cumulative particle emission rate *P*_*M*_ for pre-adolescent children and compare it to data for adults. Further, we derive a particle volume rate (PVR) using the observed particle size distribution. PVR, which can be linked to viral load [11], was calculated while breathing, speaking, and singing, with data added for shouting, as the higher vocal intensity is typical of many reference situations. Based on these measures for pre-adolescent children, the relevant risk assessment and risk management strategies should be modified specifically for child voice use.

## 2. Material and methods

### 2.1. Participants

Four girls and 11 boys, all between eight and ten years old (median 9.7 years old), took part in the study. All were members of two semi-professional children’s choirs (Staats- und Domchor Berlin, Girl’s Chorus of the Singakademie zu Berlin) with singing experience ranging from 1.5 to 4.5 years. As a reference group, 15 members (eight women, seven men, aged 23 to 64 years old, median 43.1 years old) of a semi-professional adult choir (Philharmonischer Chor Berlin) were examined.

### 2.2. Particle measurements

As described previously (see [6,7] for details), particle emission rates (*P*_*M*_) were measured under clean room conditions at the Hermann-Rietschel-Institut at Technische Universität Berlin.

The *P*_*M*_ values were converted to PVR values taking into account the average size of each measurement channel (0.4, 0.75, 2.0, 4.0, 7.5, 17.5 μm) and assuming a spherical geometry: $\text{PVR}=\sum_{i=1}^{6}[P_{M_{i}}\cdot \pi /6\cdot {\left(R_{\mathrm{ev}}\cdot d_{i}\right)}^{3}]$.

To investigate the influence of different particle size distributions within the measurement channels, we used a Monte Carlo method with *N* = 1000 repetitions for the assumptions of uniform and lognormal distributions, respectively, of particle diameter within each size class.

In order to estimate the droplet size at the mouth, a correction factor *R*_ev_ was calculated after Netz [12] for droplet evaporation under the assumption of the presence of 0.5% non-volatile solutes and about 2 s retention time in a glass pipe using the relative humidity (RH) of the cleanroom on the day of measurement. Both RH and *R*_ev_ are provided in the electronic supplementary material.

### 2.3. Test conditions

The *P*_*M*_ and PVR of children and adults were compared for different test conditions: (a) breathing at rest, as well as (b) speaking, (c) singing, and (d) shouting as differing modes of vocalization. Test condition (b) comprised the determination of *P*_*M*_ while speaking the text ‘Seefahrt nach Rio’ by James Krüss for the children, and ‘Der Nordwind und die Sonne’ by Aesop for the adults with a moderate speaking volume. Test condition (c) comprised singing the melody ‘Freude schöner Götterfunken’ in F Major (Ludwig van Beethoven, Ode to Joy, 9th Symphony in D Minor, op. 125) at moderate loudness, a familiar piece for both the child and adult subjects. For test condition (d), the subjects were asked to count ascending without pause in a loud shout. The entire duration for a measured sequence amounted to 30 s for the test conditions (a), (b), and (c) and 10 s for test condition (d). Each test condition was carried out five times. The emission rates were normalized to the respective durations of the test conditions (10 or 30 s) and therefore represent time-averaged values. The maximum sound pressure levels $L_{{\mathrm{AF}}_{\max}}$ were recorded for all measurements within each test condition.

### 2.4. Statistical analysis

The statistical analyses were conducted through linear mixed-effects modeling (*lmerTest* package, v.3.1-3) with the statistics software environment R (v.4.1.1, www.r-project.org). log_10_*P*_*M*_ was chosen as the dependent variable, and the test conditions and age group were included as fixed effects. To incorporate the influence of sound pressure level, we added $L_{{\mathrm{AF}}_{\max}}$ as an additional fixed effect. Additionally, the ID of the subject was considered as a random effect. In a second model, log_10_PVR was chosen as the dependent variable. The *p*-values were determined using Satterthwaite approximation.

## 3. Results

**Figure 1.**
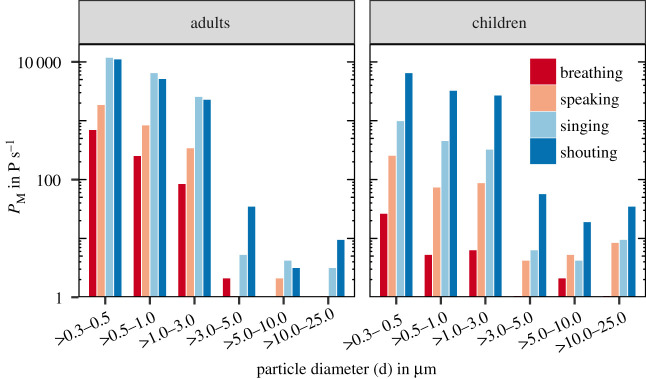
Representation of the particle distributions for size classes up to 25 μm normalized to the number of participants in each age group and the number of repetitions. We observed similar distributions for both adults (left) and children (right). Children emitted fewer particles than adults.

Similar size distributions of *P*_*M*_ were observed for both children and adults. Moreover, the shape of the size distributions is nearly independent of the test conditions and skewed to the small values (figure 1).

**Figure 2.**
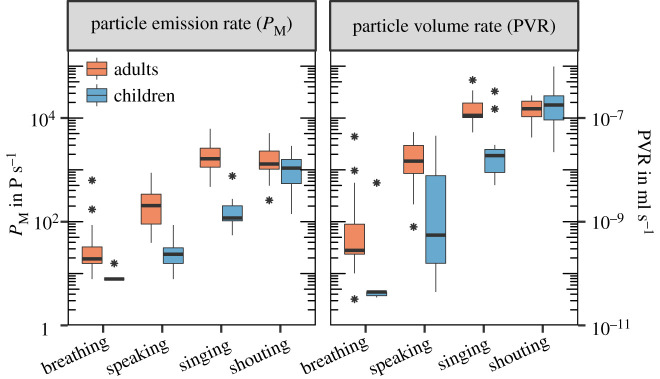
Comparison of particle emission rate in particles per second (left) and the particle volume rate in millilitres per second (right) for breathing at rest and the different vocalization types speaking, singing and shouting. The box plots are based on the distributions of individual medians of five repetitions per test condition for children (blue, *n* = 15) and adults (orange, *n* = 15).

**Figure 3.**
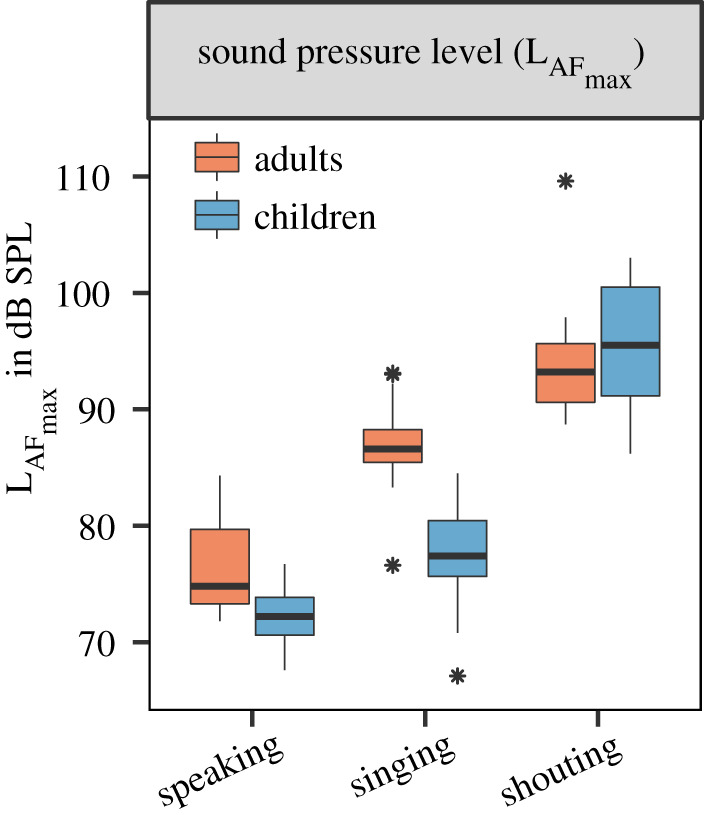
Comparison of $L_{{\mathrm{AF}}_{\max}}$ for the different vocalization types speaking, singing and shouting. The box plots are based on the distributions of individual medians of five repetitions per test condition for children (blue, *n* = 15) and adults (orange, *n* = 15). The lower limit of the *y*-axis is equivalent to the maximum value for ambient noise at 64.1 dB SPL.

For the initial descriptive analysis, we analysed the individual medians, considering only the non-zero values of participants in each group for the separate repetitions of each test condition. For breathing at rest, the median cumulative *P*_*M*_ of all examined children was 8 P s^−1^ (particles per second) (PVR = 4.4 × 10^−11^ ml s^−1^). For the different vocalization types speaking, singing and shouting, the median *P*_*M*_ were 24 P s^−1^, 118 P s^−1^ and 1083 P s^−1^, respectively (PVRs of 5.6 × 10^−10^ ml s^−1^, 1.9 × 10^−8^ ml s^−1^ and 1.8 × 10^−7^ ml s^−1^). Within the adult reference group, the median of the cumulative *P*_*M*_ for breathing at rest was 20 P s^−1^ (PVR of 2.8 × 10^−10^ ml s^−1^). For the different vocalization types speaking, singing and shouting, the median *P*_*M*_ were 204 P s^−1^, 1640 P s^−1^ and 1295 P s^−1^, respectively (PVRs of 1.5 × 10^−8^ ml s^−1^, 1.1 × 10^−7^ ml s^−1^ and 1.5 × 10^−7^ ml s^−1^) (figure 2). Thus, the *P*_*M*_ decreased from adults to children for breathing at rest, speaking, singing and shouting by factors of 2.5, 8.7, 13.9 and 1.2, respectively. Further, the PVR decreased from adults to children for breathing at rest, speaking and singing by a factor of 6.4, 26.5, 6.0, while increasing for shouting by a factor of 1.2. For children, the group medians of $L_{{\mathrm{AF}}_{\max}}$ for speaking, singing and shouting were 72.2 dB SPL (ranging from 67.6 to 76.7 dB SPL), 77.4 dB SPL (ranging from 67.1 to 84.5 dB SPL), and 95.5 dB SPL (ranging from 86.2 to 103.0 dB SPL), respectively. For adults, these values were 74.8 dB SPL (ranging from 71.8 to 84.3 dB SPL), 86.6 dB SPL (ranging from 76.6 to 93.1 dB SPL) and 93.2 dB SPL (ranging from 88.7 to 109.6 dB SPL), respectively (figure 3). For breathing at rest, $L_{{\mathrm{AF}}_{\max}}$ was dominated by the ambient noise and was not analysed. Thus, $L_{{\mathrm{AF}}_{\max}}$ values decreased from adults to children for speaking and singing by a factor of 1.3 and 2.9, while increasing for shouting by a factor of 1.3.

For further analysis, we scaled both *P*_*M*_ and PVR to the minimal detectable non-zero values to avoid non-finite values during logarithmic transformation. Linear mixed-effects modelling across the entire dataset showed a significant fixed effect of age for log-transformed *P*_*M*_ and PVR. log_10_*P*_*M*_ decreased from adults to children by a factor of 0.629 (*F* = −0.629 ± 0.090, *p* < 0.001), and log_10_PVR decreased from adults to children by a factor of 0.683 (*F* = −0.683 ± 0.132, *p* < 0.001). On the linear scale, the *P*_*M*_ for the child group was therefore reduced by a factor of 4.3, and PVR was reduced by a factor of 4.8 when compared with the adult group. To quantify the effect of $L_{{\mathrm{AF}}_{\max}}$, we computed cascading linear mixed-effect models with and without considering $L_{{\mathrm{AF}}_{\max}}$ as a fixed effect. These additional models were calculated without data for breathing at rest. We found that $L_{{\mathrm{AF}}_{\max}}$ affected log_10_*P*_*M*_ ($\chi_{1}^{2}(1)=110.47,p<0.001$) and log_10_PVR ($\chi_{1}^{2}(1)=36.168,p<0.001$). An increase of $L_{{\mathrm{AF}}_{\max}}$ of 1 dB SPL increased log_10_*P*_*M*_ by a factor of 0.042 (*F* = 0.042 ±0.004, *p* < 0.001), and log_10_PVR by a factor of 0.055 (*F* = 0.055 ±0.009, *p* < 0.001). $L_{{\mathrm{AF}}_{\max}}$ as an additional fixed effect reduced the differences between age from a factor of 4.3 to 3.3 for *P*_*M*_ and from a factor of 4.8 to 2.9 for PVR.

## 4. Discussion

In this study, we measured particle emission rates for children and adults for different conditions. We did not measure particle emissions directly at the open mouth, but rather determined them indirectly assuming homogeneously distributed particles at the probe location [6,7]. Therefore, we might not have detected all emitted particles; especially, large particles greater than 10 μm may have sedimented before entering the measurement device. However, our determined *P*_*M*_ values represent an average sample rate for each measurement and participant and allow relative comparison between the test conditions.

Similar to previous studies of particle emissions during speaking and singing, we observed large intersubject variability in both child and adult groups. Compared with adults, children’s *P*_*M*_ and PVR were both substantially lower for breathing at rest, speaking and singing conditions. More precisely, children emitted *P*_*M*_ and PVR during speaking of the same order of magnitude as adults breathing, and while singing, they emitted similar *P*_*M*_ and PVR to the adults while speaking. For the speaking condition, the text passage read by the children differed from that of adults. We suspect that this is not responsible for observed differences between the age groups as the sung *P*_*M*_ values for the adult group were similar to those derived for professional singers previously [6] although the groups performed different pieces. For the test conditions speaking and singing, we asked the children and adults to complete the task at a moderate loudness. Our determined values for $L_{{\mathrm{AF}}_{\max}}$, limited to the maximum for each measurement, do not cover the dynamic range within one repetition. We can show that about 23% of the variation in *P*_*M*_ and about 40% in PVR between children and adults are explained by $L_{{\mathrm{AF}}_{\max}}$. Therefore, the lower sound intensity of the child group only partially explains the observed difference in these measures. Further causes can be inferred from the anatomical and physiological differences between child and adult voice production. Children have shorter vocal folds and demonstrate shorter contact times during the oscillation cycles of a child’s vocal register. Age dependency of *P*_*M*_ has been demonstrated previously in a study with teenagers, whose emission rates lay between the emission rates of adults and the pre-adolescent children measured in this study [7]. The comparison of particle emissions between different test conditions, especially between speaking and singing, confirmed the higher particle emission rates for singing described among teenagers and adults.

The chosen experimental method for the determination of *P*_*M*_ particle emissions using a laser particle counter in a clean room has been shown to count the number of particles at the probe location in equilibrium after vaporization with a high level of accuracy but does not directly allow for statements regarding diameter and concentration of the emitted particles at the mouth. Consequently, PVR was estimated considering the relative humidity in the measurement room [12].

To quantify the uncertainty in PVR calculations using the mean diameters of each size class, we used a Monte Carlo method to simulate multiple repetitions of our measurements. Based on this approach, we can report that the increase of PVR from children to adults changes from 4.8 to 5.0 under the assumption of uniformly distributed particle diameters. For lognormal distributed diameters, this factor changes to 3.7. One reason for that variability is the low numbers of particles found in the size class >10−25 μm. This might be influenced by sedimentation effects within the glass pipe and, more importantly, the lower prevalence of large particles during phonatory activities. A more exact determination of these large particles would be made possible by a longer measurement time, which was not actionable within this study [13].

The lower *P*_*M*_ and PVR among children, when compared with adults, should be discussed concerning the prevalence of SARS-CoV-2 infections. Recent data from PCR measurements suggest that younger children have similar viral load when compared with adults [11]. Therefore, our data might serve as a basis for comparisons between the age groups, assuming an identical virus concentration in aerosol particles as in sputum.

However, generalizing conclusions from age comparisons should be drawn very carefully, recognizing the high person-to-person variability in emitted particles and viral loads in the case of an infection [14]. It should be emphasized that we determined data for individuals not infected with SARS-CoV-2. Further investigations are necessary to evaluate particle emissions for infected children with and without symptoms.

Our data, determined for pre-adolescent children under example test conditions, should be included in further specifications of risk assessment and hygiene concepts for schools and extracurricular activities. In this way, the risk of infection can be estimated comprehensively for different constellations in these environments based on infection risk models [15,16]. Although our data show an overall decrease in particle emission in non-infected pre-adolescent children by a factor of 4.3 in *P*_*M*_, the inter-individual viral loads span a range of several orders of magnitude.

Therefore, these studies do not allow for a generalized, less stringent risk management for children. Especially in constellations with high vocal loudness, which are common in the everyday life of children and adolescents, the aerosol emission could be similar for different age groups, as the measurements for the shouting condition show. On the other hand, singing and talking at volumes typical for children might be less restricted if the time frame is limited like in common school and extracurricular settings. However, many other factors can also play a role, such as the number of children and the design of indoor ventilation. Therefore, additional research is needed to establish further specifications of valid recommendations for school activities to provide children access to the best possible education and social participation.

## Data accessibility

Raw data, data analysis and statistics are part of the submission and provided in the electronic supplementary material. All scripts used in this study are openly accessible through https://github.com/StochasticBiology/boolean-efflux.git. The data are provided in electronic supplementary material [17].
